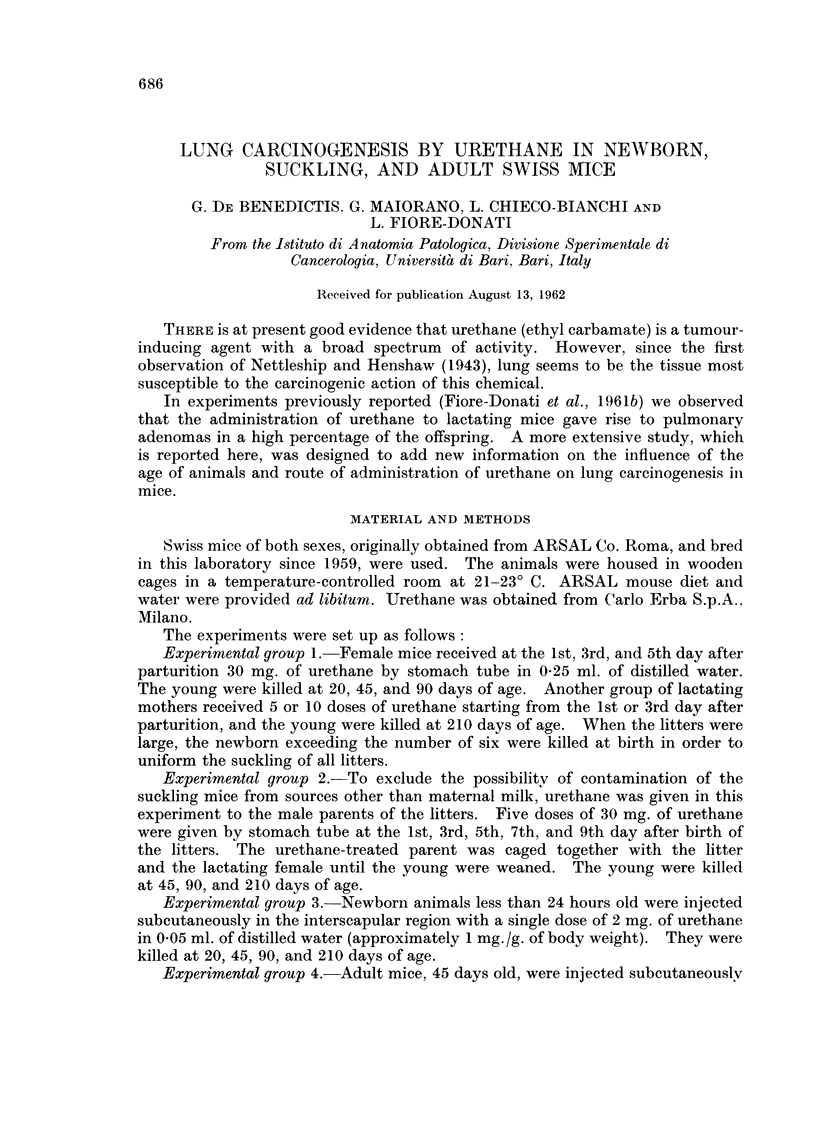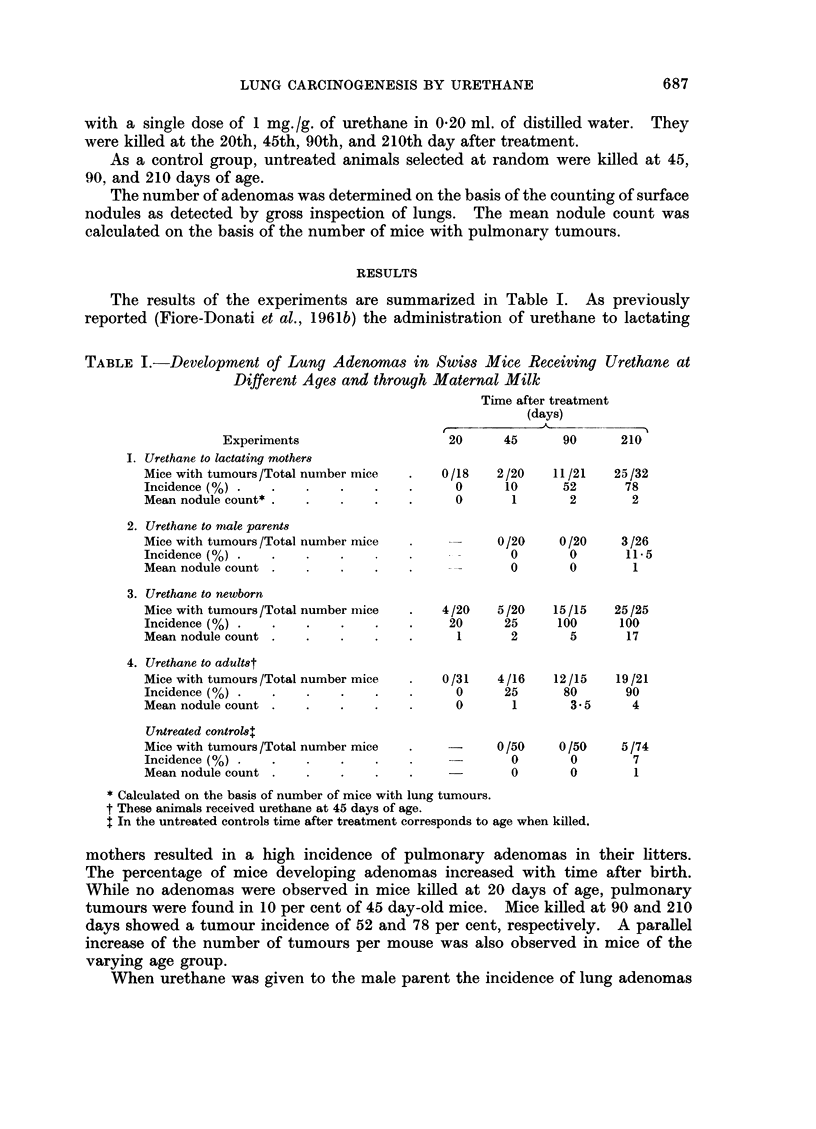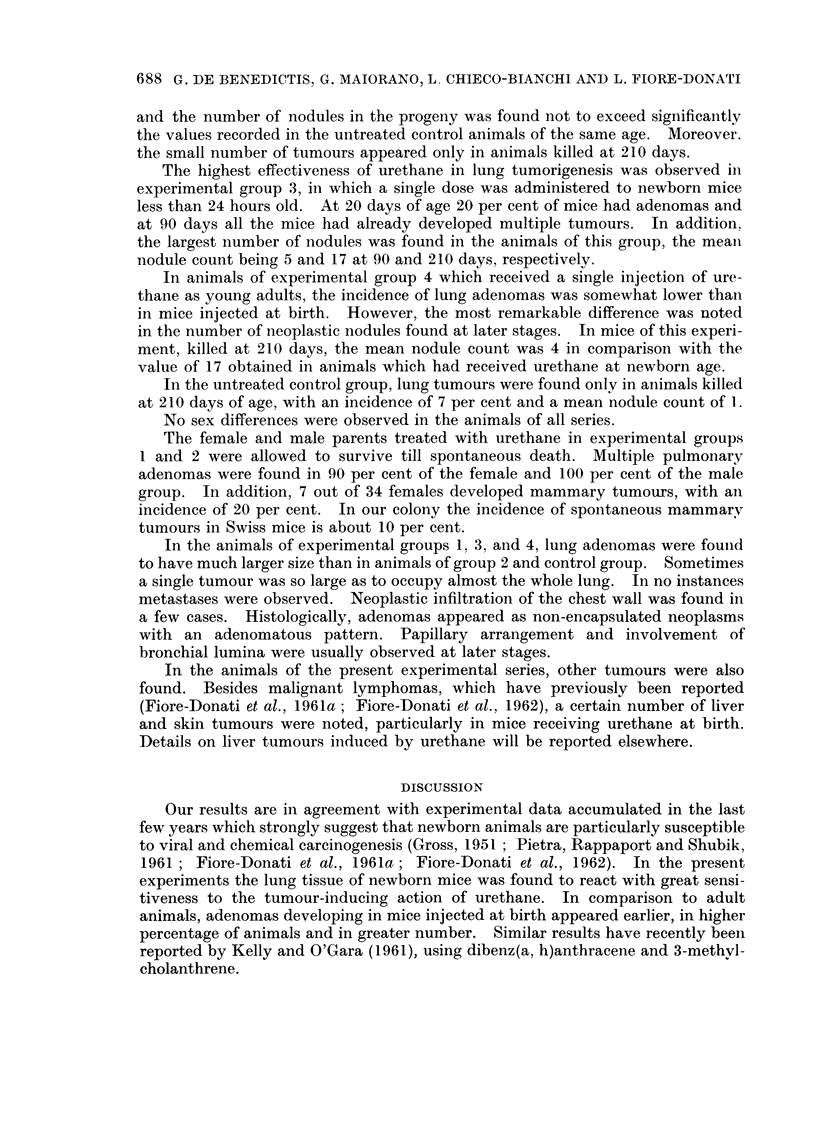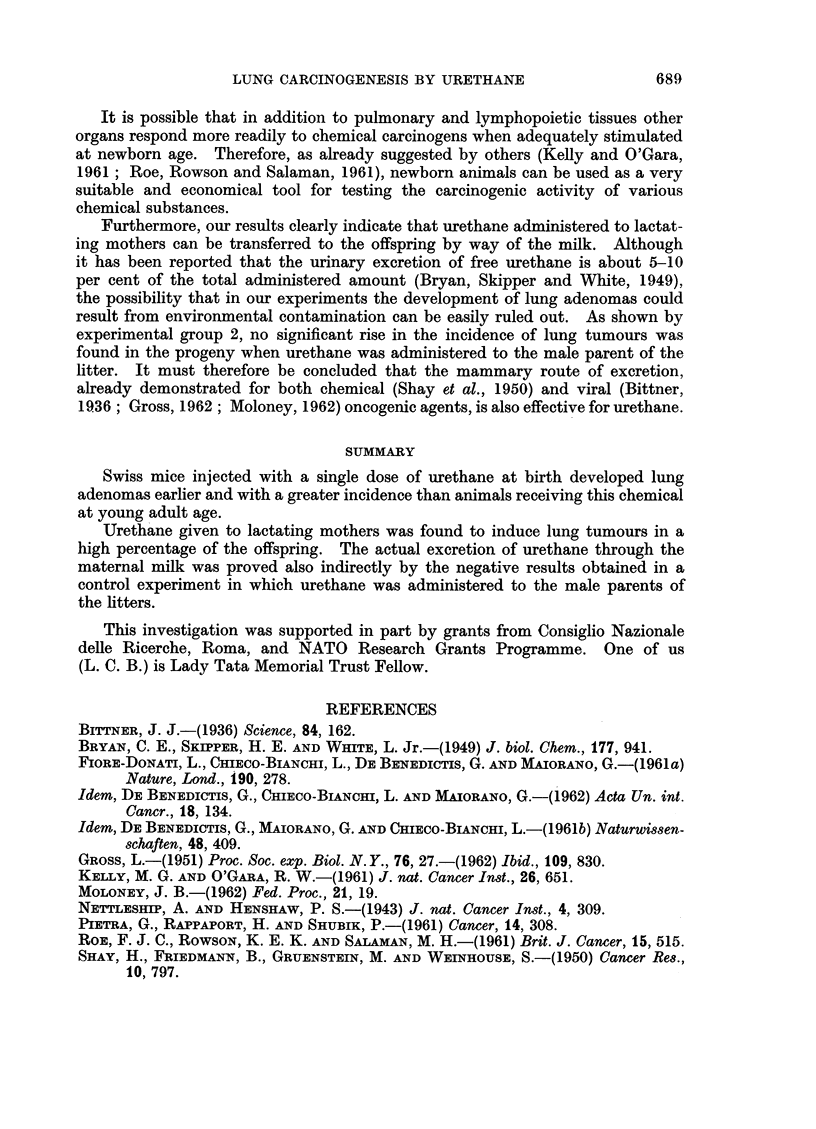# Lung Carcinogenesis by Urethane in Newborn, Suckling, and Adult Swiss Mice

**DOI:** 10.1038/bjc.1962.78

**Published:** 1962-12

**Authors:** G. De Benedictis, G. Maiorano, L. Chieco-Bianchi, L. Fiore-Donati


### LUNG CARCINOGENESIS BY URETHANE IN NEWBORN, SUCKLING, AND ADULT SWISS MICE

G. DE BENEDICTIS, G. MAIORANO, L. CHIECO-BIANCHI AND L. FIORE-DONATI

*From the Istituto di Anatomia Patologica, Divisione Sperimentale di Cancerologia, Università di Bari, Bari, Italy*

Received for publication August 13, 1962

THERE is at present good evidence that urethane (ethyl carbamate) is a tumour-inducing agent with a broad spectrum of activity. However, since the first observation of Nettleship and Henshaw (1943), lung seems to be the tissue most susceptible to the carcinogenic action of this chemical.

In experiments previously reported (Fiore-Donati *et al.*, 1961*b*) we observed that the administration of urethane to lactating mice gave rise to pulmonary adenomas in a high percentage of the offspring. A more extensive study, which is reported here, was designed to add new information on the influence of the age of animals and route of administration of urethane on lung carcinogenesis in mice.

### MATERIAL AND METHODS

Swiss mice of both sexes, originally obtained from ARSAL Co. Roma, and bred in this laboratory since 1959, were used. The animals were housed in wooden cages in a temperature-controlled room at 21–23° C. ARSAL mouse diet and water were provided *ad libitum*. Urethane was obtained from Carlo Erba S.p.A., Milano.

The experiments were set up as follows :

*Experimental group* 1.—Female mice received at the 1st, 3rd, and 5th day after parturition 30 mg. of urethane by stomach tube in 0·25 ml. of distilled water. The young were killed at 20, 45, and 90 days of age. Another group of lactating mothers received 5 or 10 doses of urethane starting from the 1st or 3rd day after parturition, and the young were killed at 210 days of age. When the litters were large, the newborn exceeding the number of six were killed at birth in order to uniform the suckling of all litters.

*Experimental group* 2.—To exclude the possibility of contamination of the suckling mice from sources other than maternal milk, urethane was given in this experiment to the male parents of the litters. Five doses of 30 mg. of urethane were given by stomach tube at the 1st, 3rd, 5th, 7th, and 9th day after birth of the litters. The urethane-treated parent was caged together with the litter and the lactating female until the young were weaned. The young were killed at 45, 90, and 210 days of age.

*Experimental group* 3.—Newborn animals less than 24 hours old were injected subcutaneously in the interscapular region with a single dose of 2 mg. of urethane in 0·05 ml. of distilled water (approximately 1 mg./g. of body weight). They were killed at 20, 45, 90, and 210 days of age.

*Experimental group* 4.—Adult mice, 45 days old, were injected subcutaneously with a single dose of 1 mg./g. of urethane in 0·20 ml. of distilled water. They were killed at the 20th, 45th, 90th, and 210th day after treatment.

As a control group, untreated animals selected at random were killed at 45, 90, and 210 days of age.

The number of adenomas was determined on the basis of the counting of surface nodules as detected by gross inspection of lungs. The mean nodule count was calculated on the basis of the number of mice with pulmonary tumours.

### RESULTS

The results of the experiments are summarized in Table I. As previously reported (Fiore-Donati *et al.*, 1961*b*) the administration of urethane to lactating

TABLE I.—*Development of Lung Adenomas in Swiss Mice Receiving Urethane at Different Ages and through Maternal Milk*

| Experiments | Time after treatment (days) 20 | 45 | 90 | 210 |
|---|---|---|---|---|
| I. *Urethane to lactating mothers* | | | | |
| Mice with tumours/Total number mice | 0/18 | 2/20 | 11/21 | 25/32 |
| Incidence (%) | 0 | 10 | 52 | 78 |
| Mean nodule count* | 0 | 1 | 2 | 2 |
| 2. *Urethane to male parents* | | | | |
| Mice with tumours/Total number mice | — | 0/20 | 0/20 | 3/26 |
| Incidence (%) | — | 0 | 0 | 11·5 |
| Mean nodule count | — | 0 | 0 | 1 |
| 3. *Urethane to newborn* | | | | |
| Mice with tumours/Total number mice | 4/20 | 5/20 | 15/15 | 25/25 |
| Incidence (%) | 20 | 25 | 100 | 100 |
| Mean nodule count | 1 | 2 | 5 | 17 |
| 4. *Urethane to adults*† | | | | |
| Mice with tumours/Total number mice | 0/31 | 4/16 | 12/15 | 19/21 |
| Incidence (%) | 0 | 25 | 80 | 90 |
| Mean nodule count | 0 | 1 | 3·5 | 4 |
| *Untreated controls*‡ | | | | |
| Mice with tumours/Total number mice | — | 0/50 | 0/50 | 5/74 |
| Incidence (%) | — | 0 | 0 | 7 |
| Mean nodule count | — | 0 | 0 | 1 |

* Calculated on the basis of number of mice with lung tumours.
† These animals received urethane at 45 days of age.
‡ In the untreated controls time after treatment corresponds to age when killed.

mothers resulted in a high incidence of pulmonary adenomas in their litters. The percentage of mice developing adenomas increased with time after birth. While no adenomas were observed in mice killed at 20 days of age, pulmonary tumours were found in 10 per cent of 45 day-old mice. Mice killed at 90 and 210 days showed a tumour incidence of 52 and 78 per cent, respectively. A parallel increase of the number of tumours per mouse was also observed in mice of the varying age group.

When urethane was given to the male parent the incidence of lung adenomas and the number of nodules in the progeny was found not to exceed significantly the values recorded in the untreated control animals of the same age. Moreover, the small number of tumours appeared only in animals killed at 210 days.

The highest effectiveness of urethane in lung tumorigenesis was observed in experimental group 3, in which a single dose was administered to newborn mice less than 24 hours old. At 20 days of age 20 per cent of mice had adenomas and at 90 days all the mice had already developed multiple tumours. In addition, the largest number of nodules was found in the animals of this group, the mean nodule count being 5 and 17 at 90 and 210 days, respectively.

In animals of experimental group 4 which received a single injection of urethane as young adults, the incidence of lung adenomas was somewhat lower than in mice injected at birth. However, the most remarkable difference was noted in the number of neoplastic nodules found at later stages. In mice of this experiment, killed at 210 days, the mean nodule count was 4 in comparison with the value of 17 obtained in animals which had received urethane at newborn age.

In the untreated control group, lung tumours were found only in animals killed at 210 days of age, with an incidence of 7 per cent and a mean nodule count of 1.

No sex differences were observed in the animals of all series.

The female and male parents treated with urethane in experimental groups 1 and 2 were allowed to survive till spontaneous death. Multiple pulmonary adenomas were found in 90 per cent of the female and 100 per cent of the male group. In addition, 7 out of 34 females developed mammary tumours, with an incidence of 20 per cent. In our colony the incidence of spontaneous mammary tumours in Swiss mice is about 10 per cent.

In the animals of experimental groups 1, 3, and 4, lung adenomas were found to have much larger size than in animals of group 2 and control group. Sometimes a single tumour was so large as to occupy almost the whole lung. In no instances metastases were observed. Neoplastic infiltration of the chest wall was found in a few cases. Histologically, adenomas appeared as non-encapsulated neoplasms with an adenomatous pattern. Papillary arrangement and involvement of bronchial lumina were usually observed at later stages.

In the animals of the present experimental series, other tumours were also found. Besides malignant lymphomas, which have previously been reported (Fiore-Donati *et al.*, 1961*a* ; Fiore-Donati *et al.*, 1962), a certain number of liver and skin tumours were noted, particularly in mice receiving urethane at birth. Details on liver tumours induced by urethane will be reported elsewhere.

### DISCUSSION

Our results are in agreement with experimental data accumulated in the last few years which strongly suggest that newborn animals are particularly susceptible to viral and chemical carcinogenesis (Gross, 1951 ; Pietra, Rappaport and Shubik, 1961 ; Fiore-Donati *et al.*, 1961*a* ; Fiore-Donati *et al.*, 1962). In the present experiments the lung tissue of newborn mice was found to react with great sensitiveness to the tumour-inducing action of urethane. In comparison to adult animals, adenomas developing in mice injected at birth appeared earlier, in higher percentage of animals and in greater number. Similar results have recently been reported by Kelly and O'Gara (1961), using dibenz(a, h)anthracene and 3-methylcholanthrene.

It is possible that in addition to pulmonary and lymphopoietic tissues other organs respond more readily to chemical carcinogens when adequately stimulated at newborn age. Therefore, as already suggested by others (Kelly and O'Gara, 1961 ; Roe, Rowson and Salaman, 1961), newborn animals can be used as a very suitable and economical tool for testing the carcinogenic activity of various chemical substances.

Furthermore, our results clearly indicate that urethane administered to lactating mothers can be transferred to the offspring by way of the milk. Although it has been reported that the urinary excretion of free urethane is about 5–10 per cent of the total administered amount (Bryan, Skipper and White, 1949), the possibility that in our experiments the development of lung adenomas could result from environmental contamination can be easily ruled out. As shown by experimental group 2, no significant rise in the incidence of lung tumours was found in the progeny when urethane was administered to the male parent of the litter. It must therefore be concluded that the mammary route of excretion, already demonstrated for both chemical (Shay *et al.*, 1950) and viral (Bittner, 1936 ; Gross, 1962 ; Moloney, 1962) oncogenic agents, is also effective for urethane.

### SUMMARY

Swiss mice injected with a single dose of urethane at birth developed lung adenomas earlier and with a greater incidence than animals receiving this chemical at young adult age.

Urethane given to lactating mothers was found to induce lung tumours in a high percentage of the offspring. The actual excretion of urethane through the maternal milk was proved also indirectly by the negative results obtained in a control experiment in which urethane was administered to the male parents of the litters.

This investigation was supported in part by grants from Consiglio Nazionale delle Ricerche, Roma, and NATO Research Grants Programme. One of us (L. C. B.) is Lady Tata Memorial Trust Fellow.

### REFERENCES

Bittner, J. J.—(1936) *Science*, **84**, 162.

Bryan, C. E., Skipper, H. E. and White, L. Jr.—(1949) *J. biol. Chem.*, **177**, 941.

Fiore-Donati, L., Chieco-Bianchi, L., De Benedictis, G. and Maiorano, G.—(1961*a*) *Nature, Lond.*, **190**, 278.

*Idem*, De Benedictis, G., Chieco-Bianchi, L. and Maiorano, G.—(1962) *Acta Un. int. Cancr.*, **18**, 134.

*Idem*, De Benedictis, G., Maiorano, G. and Chieco-Bianchi, L.—(1961*b*) *Naturwissenschaften*, **48**, 409.

Gross, L.—(1951) *Proc. Soc. exp. Biol. N.Y.*, **76**, 27.—(1962) *Ibid.*, **109**, 830.

Kelly, M. G. and O'Gara, R. W.—(1961) *J. nat. Cancer Inst.*, **26**, 651.

Moloney, J. B.—(1962) *Fed. Proc.*, **21**, 19.

Nettleship, A. and Henshaw, P. S.—(1943) *J. nat. Cancer Inst.*, **4**, 309.

Pietra, G., Rappaport, H. and Shubik, P.—(1961) *Cancer*, **14**, 308.

Roe, F. J. C., Rowson, K. E. K. and Salaman, M. H.—(1961) *Brit. J. Cancer*, **15**, 515.

Shay, H., Friedmann, B., Gruenstein, M. and Weinhouse, S.—(1950) *Cancer Res.*, **10**, 797.